# Identification and validation of Alzheimer’s disease-related metabolic brain pattern in biomarker confirmed Alzheimer’s dementia patients

**DOI:** 10.1038/s41598-022-15667-9

**Published:** 2022-07-11

**Authors:** Matej Perovnik, Petra Tomše, Jan Jamšek, Andreja Emeršič, Chris Tang, David Eidelberg, Maja Trošt

**Affiliations:** 1 Department of Neurology, University Medical Center Ljubljana, Zaloska cesta 2, 1000 Ljubljana, Slovenia; 2 Faculty of Medicine, University of Ljubljana, Vrazov trg 2, 1000 Ljubljana, Slovenia; 3 Department of Nuclear Medicine, University Medical Center Ljubljana, Zaloska cesta 2, 1000 Ljubljana, Slovenia; 4 Laboratory for CSF Diagnostics, Department of Neurology, University Medical Center Ljubljana, Zaloska cesta 2, 1000 Ljubljana, Slovenia; 5 Center for Neurosciences, The Feinstein Institutes for Medical Research, 350 Community Drive, Manhasset, NY 11030 USA

**Keywords:** Dementia, Biomarkers, Neuroscience, Alzheimer's disease

## Abstract

Metabolic brain biomarkers have been incorporated in various diagnostic guidelines of neurodegenerative diseases, recently. To improve their diagnostic accuracy a biologically and clinically homogeneous sample is needed for their identification. Alzheimer’s disease-related pattern (ADRP) has been identified previously in cohorts of clinically diagnosed patients with dementia due to Alzheimer’s disease (AD), meaning that its diagnostic accuracy might have been reduced due to common clinical misdiagnosis. In our study, we aimed to identify ADRP in a cohort of AD patients with CSF confirmed diagnosis, validate it in large out-of-sample cohorts and explore its relationship with patients’ clinical status. For identification we analyzed 2-[^18^F]FDG PET brain scans of 20 AD patients and 20 normal controls (NCs). For validation, 2-[^18^F]FDG PET scans from 261 individuals with AD, behavioral variant of frontotemporal dementia, mild cognitive impairment and NC were analyzed. We identified an ADRP that is characterized by relatively reduced metabolic activity in temporoparietal cortices, posterior cingulate and precuneus which co-varied with relatively increased metabolic activity in the cerebellum. ADRP expression significantly differentiated AD from NC (AUC = 0.95) and other dementia types (AUC = 0.76–0.85) and its expression correlated with clinical measures of global cognition and neuropsychological indices in all cohorts.

## Introduction

Alzheimer’s disease is the most common cause of dementia, pathologically characterized by deposition of misfolded proteins, amyloid-β (Aβ) and tau, leading to neuronal dysfunction and neurodegeneration^1^. Recently, new research framework was introduced defining biomarker based diagnosis. Positivity in biomarkers of amyloid (A+) and tau (T+) was proposed for diagnosing individuals with biologically defined Alzheimer’s disease in a so called A/T/N classification scheme^2^.

2-[^18^F]fluoro-2-deoxy-d-glucose positron emission tomography (2-[^18^F]FDG PET) is a widely available non-invasive brain imaging modality that provides in vivo information about synaptic activity^3^ and is a biomarker of neurodegeneration^2^. Neurodegeneration closely correlates with subject’s progressive cognitive disability in Alzheimer’s disease^4^. In clinical setting 2-[^18^F]FDG PET scans are commonly assessed visually^5^ assisted by semi-quantitative analyses^6^ and its use is recommended for diagnostic and differential diagnostic purposes in dementia syndromes^7,8^. Multivariate analysis approaches of 2-[^18^F]FDG PET scans, such as scaled subprofile model/principal component analysis (SSM/PCA), have been used in the past to derive specific disease-related patterns to improve diagnostic accuracy and provide insight into pathophysiology^9–12^. Multivariate approaches are in general advantageous over univariate models by being able to take into account interactions between voxels/brain regions and have been shown to have better sensitivity, specificity and reproducibility^13^.

Alzheimer’s disease-related pattern (ADRP) has been identified previously in four different cohorts of patients with clinically diagnosed Alzheimer’s dementia (AD)^12,14–16^. However, clinical diagnosis is not in concordance with pathological findings in around 30% of AD cases^17^ and therefore clinically defined cohorts may be heterogeneous in their underlying cause of dementia^18^. Consequentially previously identified ADRPs may not be specific enough. Cerebrospinal fluid (CSF) biomarkers closely reflect Alzheimer’s pathology^19–21^.

The aims of this study were to (i) identify ADRP in a cohort of CSF biomarker-positive AD patients; (ii) to correlate the newly identified ADRP expression with patients’ clinical measures; (iii) to validate it on independent cohorts of patients with AD, behavioral variant of frontotemporal dementia (bvFTD) and on two mild cognitive impairment (MCI) cohorts, one due to Alzheimer’s disease and one due to other causes.

## Methods

### Participants

301 subjects from three different cohorts were included in the study. To identify and internally validate ADRP, we included 63 patients who fulfilled diagnostic criteria for AD (amnestic presentation^22^), had Alzheimer (Alz) biomarker CSF profile, i.e. A+/T+/N+ or A+/T+/N−^2,22^ and underwent 2-[^18^F]FDG PET brain imaging. Additionally, we included 42 patients with MCI who had available CSF information and 2-[^18^F]FDG PET brain scans. Patients with MCI were cognitively tested by neuropsychologist, *n* = 19/42^23^, or by neurologist using MoCA test, *n* = 23/42^24,25^. We also included 15 patients with probable bvFTD with diagnosis confirmed at a follow-up visit at least 18 months after 2-[^18^F]FDG PET scanning^26^ and 41 normal controls (NCs) scanned with 2-[^18^F]FDG PET for purposes of another research project^27^. We excluded patients with structural brain lesions (e.g. tumor, stroke) or systemic condition (e.g. hypothyroidism, B_12_ deficiency) that could cause or significantly contribute to cognitive impairment. All patients and NC from identification and internal validation cohorts underwent 2-[^18^F]FDG PET brain imaging between January 2010 and April 2019 at the University Medical Center Ljubljana (UMCL), Slovenia.

For external validation, we randomly selected 60 AD patients with Alz CSF profile and 60 NC with normal (A−/T−/N−) CSF profile from Alzheimer’s disease neuroimaging initiative (ADNI) database. Further, we also analyzed a previously described cohort of 10 patients with clinically diagnosed AD (AD-NS)^28^, and 10 age-matched NC (NC-NS)^12^ from North Shore University Hospital, Manhasset, New York, USA.

#### ADRP identification group

20 AD patients (AD1) and 20 age- and sex-matched NC subjects (NC1) were analyzed for the ADRP identification. AD patients were randomly chosen from UMCL AD dataset (*n* = 63). NC1 subjects were deemed cognitively normal either by neuropsychological evaluation, *n* = 13/20^29^, or by cognitive screening with Mini-mental state examination (MMSE) and Montreal Cognitive Assessment (MoCA), *n* = 7/20^24,30^.

#### Internal validation groups

To validate the newly-identified ADRP, we analyzed data from the remaining 43 AD patients with Alz CSF profile and additional 15 bvFTD patients, 42 patients with MCI (27 with Alz CSF profile and 15 with normal or non-Alz CSF profile), and 21 NC subjects (NC2). Patients underwent clinical neurological and neuropsychological examination, cognitive assessment using MMSE^30^ and MoCA^24^ tests, as well as structural (MRI or CT) and 2-[^18^F]FDG PET brain imaging.

#### External validation groups

To externally validate ADRP, we analyzed data from ADNI database (60 AD and 60 NC) and previously described cohort from North Shore University Hospital (10 AD and 10 NC)^12^.

The ADNI (https://adni.loni.usc.edu) was launched in 2003 as a public–private partnership, led by Principal Investigator Michael W. Weiner, MD. The primary goal of ADNI has been to test whether serial MRI, PET, other biological markers, and clinical and neuropsychological assessment can be combined to measure the progression of MCI and early Alzheimer's disease.

We selected an age homogenous group of 60 AD patients^28^ with Alz CSF profile^31,32^ and 60 NC with normal CSF profile, using an in-house script. First, we randomly picked the groups, then the script was used to randomly replace individuals until the standard deviation of age did not change after 10,000 iterations.

### Neuropsychological assessment

30 patients from UMCL AD dataset underwent detailed neuropsychological assessment and data from 15 patients in whom testing was done within 1 year of 2-[^18^F]FDG PET imaging was analyzed. Repeatable Battery for the Assessment of Neuropsychological Status (RBANS), which consists of tests of immediate (List Learning and Story Memory tests) and delayed (List Recall, List Recognition, Story Recall and Figure Recall tests) memory, visuospatial/construction (Figure Copy and Line Orientation), language (Picture Naming, Semantic Fluency) and attention (Digit Span and Coding) was used for neuropsychological testing. Index score for each cognitive domain was calculated from raw results^23^.

### Structural imaging

We checked available T1 and T2-weighted MRI scans (13/20 from AD1, 29/43 from AD2, 13/27 from MCI Alz and 3/15 from MCI nonAlz). Cerebral atrophy and white matter hyperintensities were assessed using medial temporal lobe atrophy (MTA) classification scale^33^ and Fazekas scale^34^. Despite the limitation of missing MRI data, the goal of these analyses was to examine that the ADRP identification and validation groups did not differ in hippocampal atrophy and vascular burden at a group level. In patients with inaccessible MRI, we checked CT scans obtained with Siemens Biograph mCT PET/CT scanner for structural changes.

### Cerebrospinal fluid analysis

Patients underwent lumbar puncture within 4 years of 2-[^18^F]FDG PET scanning. CSF samples were centrifuged, aliquoted and stored at −80 °C until biomarker analysis was performed at the Department of Neurology, UMCL. CSF Aβ_42_, phosphorylated tau (p-tau) and total tau protein (t-tau) were measured routinely, whereas Aβ_40_ was determined additionally in the CSF samples with ambiguous Aβ_42_ result (650–815 pg/ml). Biomarker analyses were performed according to manufacturers’ instructions using Innotest (Fujirebio, Europe) immunoassays with intra-assay variability < 5%. Between-assay coefficients of variation for Aβ_42_, Aβ_40_, p-tau and t-tau were 5.8%, 8.3%, 4.4% and 8.2%, respectively, as determined by the longitudinal quality control sample. Locally validated biomarker cut-off levels were used to define Alz CSF profile^31^ as: Aβ_42_ < 650 pg/ml or Aβ_42_/Aβ_40_ < 0.077 (A+), p-tau > 60 pg/ml (T+) and t-tau > 400 pg/ml (N+).

### 2-[^18^F]FDG PET image acquisition

2-[^18^F]FDG PET brain images of patients and NC in identification and internal validation groups were acquired at the Department of Nuclear Medicine, UMCL with Siemens Biograph mCT PET/CT scanner according to clinical diagnostic protocol^5^ as described previously^27^. Patients from ADNI cohort underwent imaging using different scanners at different sites as per study protocol, described in more detail at: https://adni.loni.usc.edu/methods/pet-analysis-method/pet-analysis/. 2-[^18^F]FDG PET brain images of patients and NC from North Shore University Hospital were acquired with GE Advance tomograph at The Feinstein Institutes for Medical Research as described previously^12^.

#### Image pre-processing

2-[^18^F]FDG PET scans were pre-processed with SPM12 (Wellcome Trust Centre for Neuroimaging, Institute of Neurology, London, UK) running on Matlab R2019a (Mathworks Inc., Natick, MA) using an in-house pipeline. 2-[^18^F]FDG PET scans from NS were pre-processed with SPM5 running on Matlab 6.0. First step was rigid registration of the scans to the template, which was followed by spatial normalization to the Montreal Neurological Institute standard space using a PET template and old normalization function. Lastly, the images were smoothed with isotropic 3D Gaussian kernel of 10 mm FWHM.

#### Image analysis

For ADRP identification SSM/PCA (ScAnVP, Center for Neuroscience, Feinstein Institutes for Medical Research, NY, USA) was applied to 2-[^18^F]FDG PET scans of 20 AD1 and 20 NC1 subjects as described previously^9,13^. The number of 20 diseased and healthy have been shown optimal in previous studies^15,35^. Further analysis was limited to principal components (PCs) that each accounted for at least 5% of the total variance (VAF). Subject scores for these PCs were then entered into a series of logistic regression models, with group as dependent and subject scores as the independent variable. The model with the lowest Akaike Information Criterion score was selected as the ADRP. Estimated disease-related metabolic pattern was tested for reliability using bootstrap resampling with 1000 iterations^36^. Pattern stability was assessed also with threefold cross-validation procedure using the data from internal validation group (AD2 and NC2). For the calculation of pattern expression, i.e. subject score, in 2-[^18^F]FDG PET images from subjects not included in the identification cohort topographic profile rating (TPR) was used^9^. In TPR, logarithmically transformed and double centered subject vectors were multiplied by the ADRP. Raw scores were Z-transformed based on the mean pattern expression and standard deviation of subject scores in the NC1 group.

### Statistical analysis

Data distribution was tested for normality using Shapiro–Wilk test. Student’s independent-sample t-test or one-way analysis of variance (ANOVA) with post hoc Tukey HSD test was used to examine differences in age, MMSE, MoCA, disease duration, MTA scores and Fazekas scores in the NC and patient groups. Fisher’s Exact Test for Count Data was used to examine differences in sex distribution. For the non-normally distributed data, the non-parametric tests (e.g. Wilcoxon-rank sum test, Spearman’s rank correlation and Kruskal Wallis test with post hoc Dunn’s test) were additionally performed to examine whether the significant results and statistical inferences of the corresponding parametric tests were changed.

To examine differences between normalized ADRP subject scores in the pattern identification group (AD1 and NC1), we used a robust exact Fisher-Pitman permutation test, which is data-dependent and free of assumptions of underlying distribution^37^. Correlations between ADRP expression and MMSE, MoCA, disease duration and neuropsychological scores were evaluated with Pearson correlation analysis. One-way ANOVA with post hoc Tukey HSD test was used to examine differences in ADRP expression in two internal dementia groups (AD2, FTD) and NC2 and between MCI Alz, MCI nonAlz and NC2 groups. We used Student’s independent-sample t-test to examine the differences in pattern expression scores between NC1 and NC2, between AD1 and AD2 and when comparing AD1 and AD2 to MCI Alz groups and external dementia validation groups from ADNI and NS. We used *pROC* package to calculate area under the curve (AUC), specificity and sensitivity based on the optimal cut-point determined by Youden’s index for the internal validation group (AD2 vs NC2)^38^. All statistical analyses were performed in RStudio version 1.3.1093, R version 3.6.0 (R Foundation for Statistical Computing, Vienna, Austria) and results were considered significant at *p* < 0.05 (two-tailed).

### Ethics approval and consent to participate

The study was approved by Slovenian National Ethics Committee (0120-584/2019/5) and institutional review boards of collaborating institutions. All patients gave informed consent. The study was designed and conducted in accordance with the relevant guidelines and regulations of the ethical principles for medical research involving human subjects﻿﻿.

## Results

Subjects’ demographic and clinical data and the results of visual assessment of structural imaging are presented in Table 1. Mean (± SD) duration between 2-[^18^F]FDG PET and lumbar puncture was 7.5 ± 10 months in UMCL and 0.5 ± 1 month ADNI dataset; and 11 ± 13 months between 2-[^18^F]FDG PET and structural imaging. MCI groups are presented in the Supplementary Material.

**Table 1:** Demographic and clinical data in identification and validation groups.

	Identification		Internal validation			External validation				*p* value
	NC1	AD1	NC2	AD2	bvFTD	NC-ADNI	AD-ADNI	NC-NS	AD-NS	
N	20	20	21	43	15	60	60	10	10	
Age (y)	68 (6.5)	72.6 (8.5)	62.6 (6.6)	73 (9)	68 (11.3)	75.0 (3.9)	76.4 (5.1)	73.4 (4.8)	74.5 (5.3)	< 0.001
Sex (m/f)	8/12	9/11	5/16	23/20	6/9	31/29	38/22	4/6	6/4	0.118
Disease duration (y)	–	4 (2.7)	–	3.3 (2) (*n* = 34)	3.3 (1.3) (*n* = 11)	–	5.2 (2.9)	–	3.8 (3.2) (*n* = 8)	0.010
MMSE	28.7 (1.1)	18 (5.8)	29.4 (0.8) (*n* = 12)	18 (4.8) (*n* = 39)	19.8 (5.2) (*n* = 13)	29.0 (1.2)	23.5 (2)	–	23.9 (4.2)	< 0.001
MoCA	26.7 (2.2)	19.8 (2.5) (*n* = 4)	27.6 (1.7) (*n* = 13)	16.1 (5.3) (*n* = 11)	14 (5.5) (*n* = 4)	25.8 (2.2) (*n* = 41)	17.0 (4.9) (*n* = 31)	–	–	< 0.001
MTA score (left + right)	–	3.8 (1.4) (*n* = 13)	/	3.4 (1.7) (*n* = 27)	–	–	–	–	–	0.371
Fazekas score	–	1 (0.6) (*n* = 13)	/	0.9 (0.6) (*n* = 29)	–	–	–	–	–	0.629
Alz CSF (positive/total)	0/10	20/20	0/3	43/43	0/10	0/60	60/60	–	–	

Data is presented as mean (SD).

Positive Alzheimer (Alz) CSF was defined as A+/T+/N+ or A+/T+/N− (cut-offs: Aβ_42_ < 650 pg/ml or Aβ_42_/Aβ_40_ < 0.077, p-tau > 60 pg/ml, t-tau > 400 pg/ml) for identification and internal validation groups and as A+/T+/N+ or A+/T+/N− (cut-offs: Aβ_42_ < 880 pg/ml or Aβ_42_/Aβ_40_ < 0.077, p-tau > 21.8 pg/ml and t-tau > 245 pg/ml) for ADNI cohort.

*NC* normal control, *AD* Alzheimer’s dementia, *bvFTD* behavioral variant of frontotemporal dementia, *NS* Northshore, *ADNI* Alzheimer’s Disease Neuroimaging Initiative, *MMSE* Mini Mental State Examination, *MoCA* Montreal Cognitive Assessment, *MTA* medial temporal lobe atrophy, *CSF* cerebrospinal fluid.

### ADRP identification

AD1 and NC1 participants did not differ in mean age (*p* = 0.44) or sex distribution (*p* = 0.09). AD1 had significantly lower MMSE scores than NC1, *p* < 0.001.

**Figure 1:**
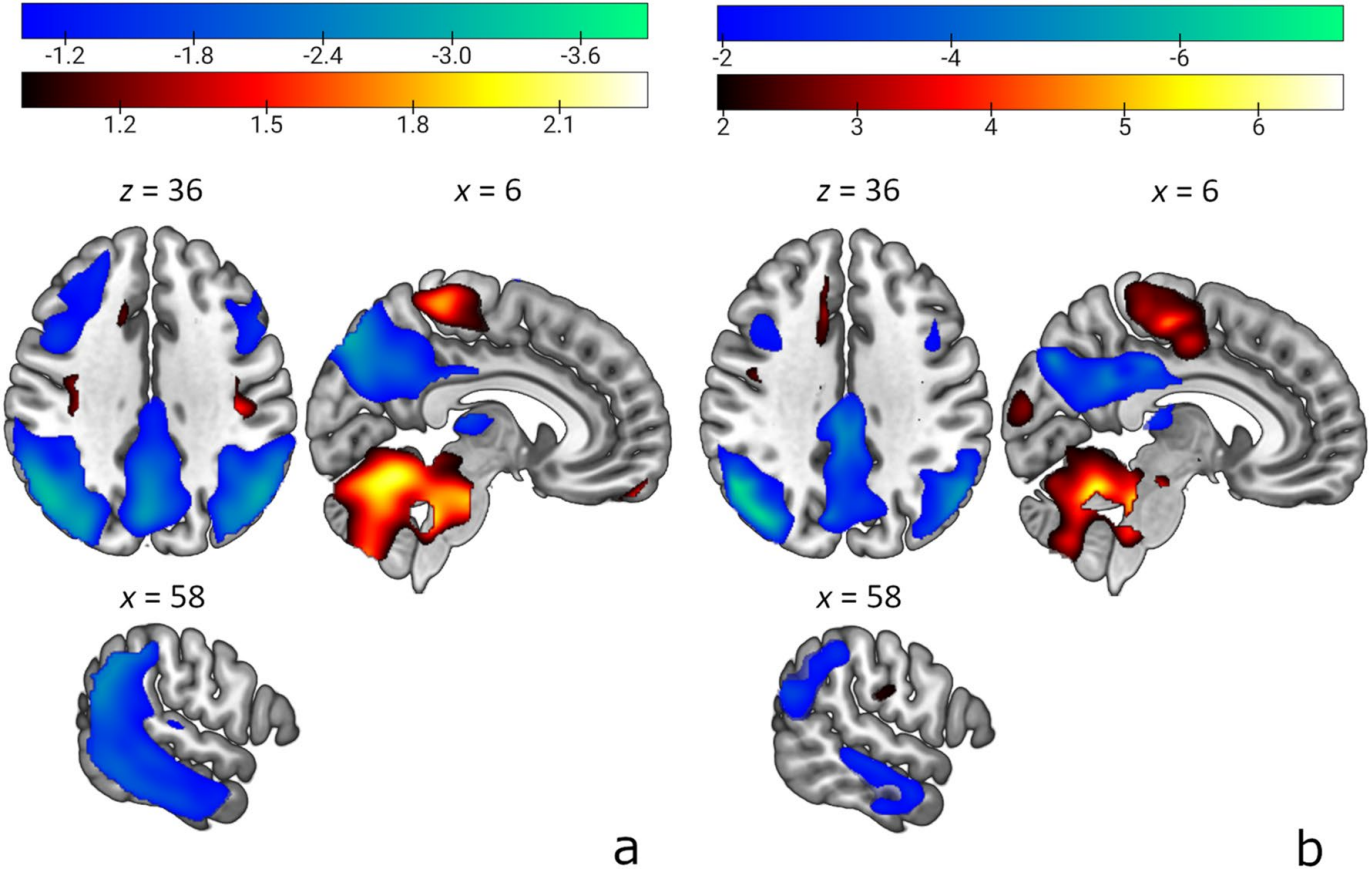
Alzheimer’s disease-related pattern (ADRP) and maps of inverse coefficient of variation overlaid on T1-weighted magnetic resonance template images. (**a**) The ADRP was characterized by relatively reduced metabolic activity (color-coded green to blue) in temporoparietal cortices, thalami, posterior cingulate and precuneus which co-varied with relatively increased metabolic activity (color-coded red to yellow) in the cerebellum. (**b**) The topography of ADRP was reliable as estimated by a voxel-wise bootstrapping algorithm. The display represents the maps of inverse coefficient of variation thresholded at z =|1.96|, p < 0.05.

Four principal components: PC1 (28.7% VAF), PC2 (9.6% VAF), PC3 (6.7% VAF) and PC4 (5.4% VAF) were entered into a series of logistic regression models for further analysis. Model that incorporated PC1 alone was determined as the best to discriminate NC1 from AD1. The ADRP was characterized by relatively reduced metabolic activity in temporoparietal cortices, posterior cingulate, thalami and precuneus which co-varied with relatively increased metabolic activity in the cerebellum, Fig. 1a. Bootstrapping proved pattern stability at *z* =|1.96|, *p* < 0.05, Fig. 1b. Cross-validation procedure showed strong and significant correlations between the topography of the three patterns (see Supplementary Information). All subsequent analyses were done using ADRP obtained with data from AD1 and NC1 participants.

**Figure 2:**
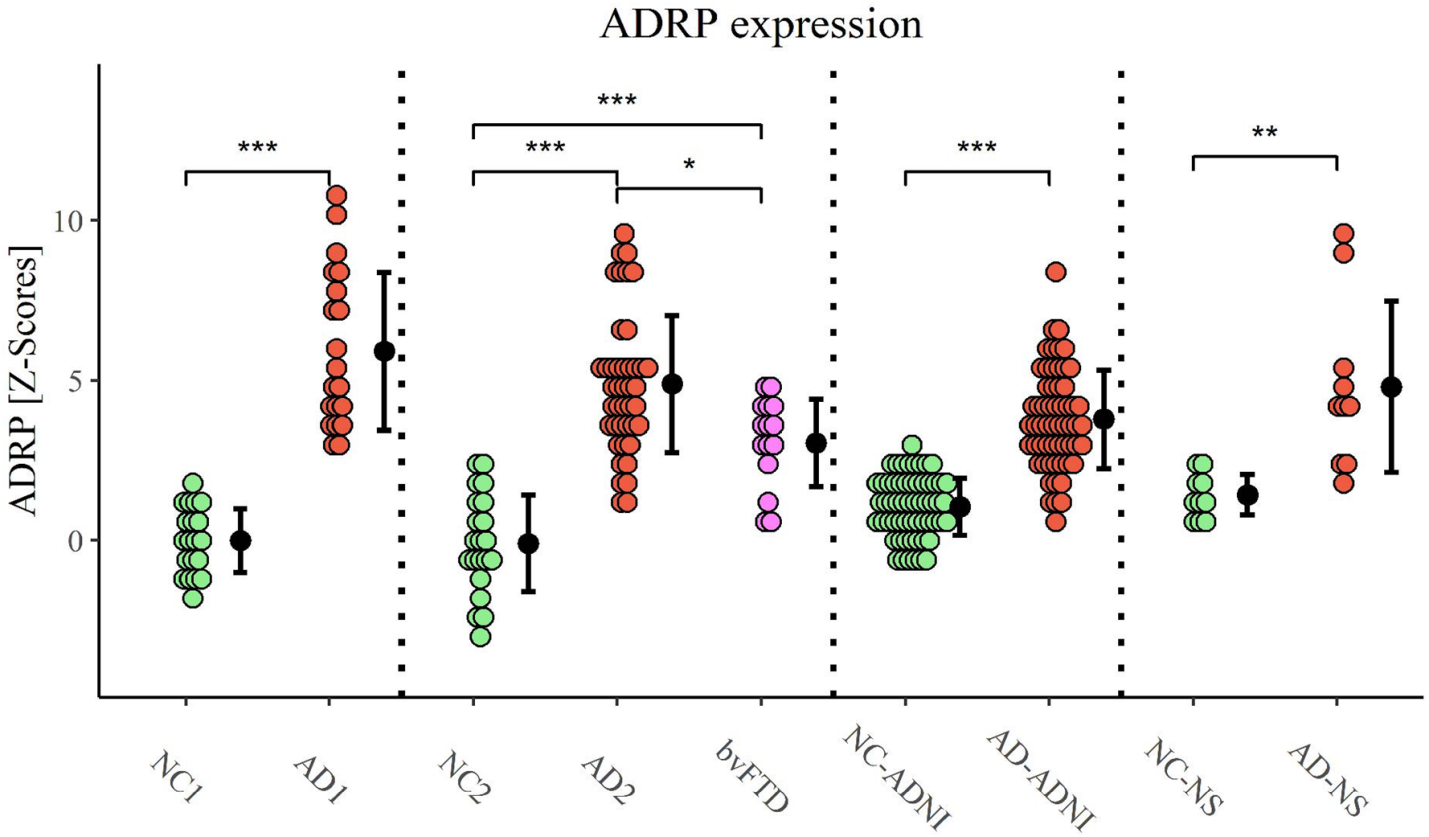
Alzheimer’s disease-related pattern expression. Data are Z-scored based on the pattern expression in NC1 group. Means (SD) are presented to the right of individual data. *NC1* normal control identification group, *AD1* Alzheimer’s dementia identification group, *NC2* NC validation group, *AD2* AD validation group, *bvFTD* behavioral variant of frontotemporal dementia, *ADNI* Alzheimer’s Disease Neuroimaging Initiative, *NS* Northshore, *ADRP* Alzheimer’s disease-related pattern. *p < 0.01, **p < 0.001, ***p < 0.0001.

**Figure 3:**
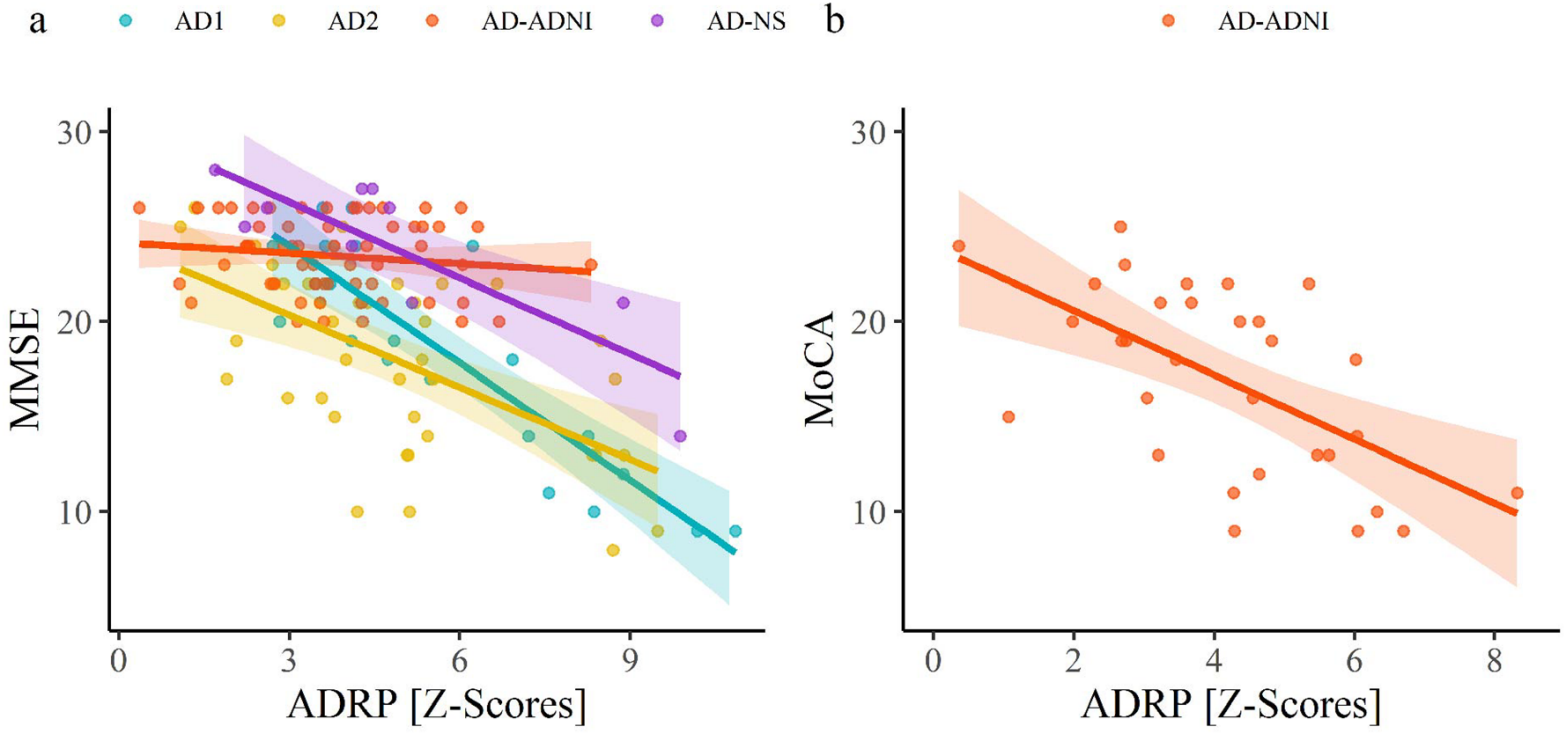
Correlation between MMSE and ADRP expression (**a**) and between MoCA and ADRP expression (**b**). Each dot represents an individual patient’s data and the lines and shaded areas correspond to the fit of a linear regression with 95% confidence intervals. (**a**) ADRP expression correlated with MMSE in AD1 (r =  − 0.88), AD2 (r =  − 0.58) and AD-NS (r =  − 0.85), but not in AD-ADNI (r =  − 0.14) group. (**b**) ADRP expression correlated with MoCA in AD-ADNI (r =  − 0.60) group. *AD1* Alzheimer’s dementia identification group, *AD2* Alzheimer’s dementia validation group, *ADNI* Alzheimer’s Disease Neuroimaging Initiative, *NS* Northshore, *ADRP* Alzheimer’s disease-related pattern, *MMSE* Mini Mental State Examination, *MoCA* Montreal Cognitive Assessment.

Pattern expression was significantly higher in AD1 (*M* = 5.9, *SD* = 2.5) compared to NC1 subjects (*M* = 0.0, *SD* = 1.0), *Z* = 5.3, *p* < 0.001, Fig. 2. ADRP subject scores strongly inversely correlated with MMSE scores in AD1 group (*r*(18) =  − 0.88, *p* < 0.001), Fig. 3a, but not with disease duration (*r*(18) = 0.07, *p* = 0.78).

### Internal ADRP validation

There was a significant difference in age between the three validation groups (*F*(2, 76) = 9.7, *p* < 0.001) and no difference in sex distribution. Post hoc comparisons showed that mean age was significantly higher in AD2 compared to NC2 (*p* < 0.001), but no difference was found between AD2 and bvFTD (*p* = 0.16) or bvFTD and NC2 groups (*p* = 0.18).

**Figure 4:**
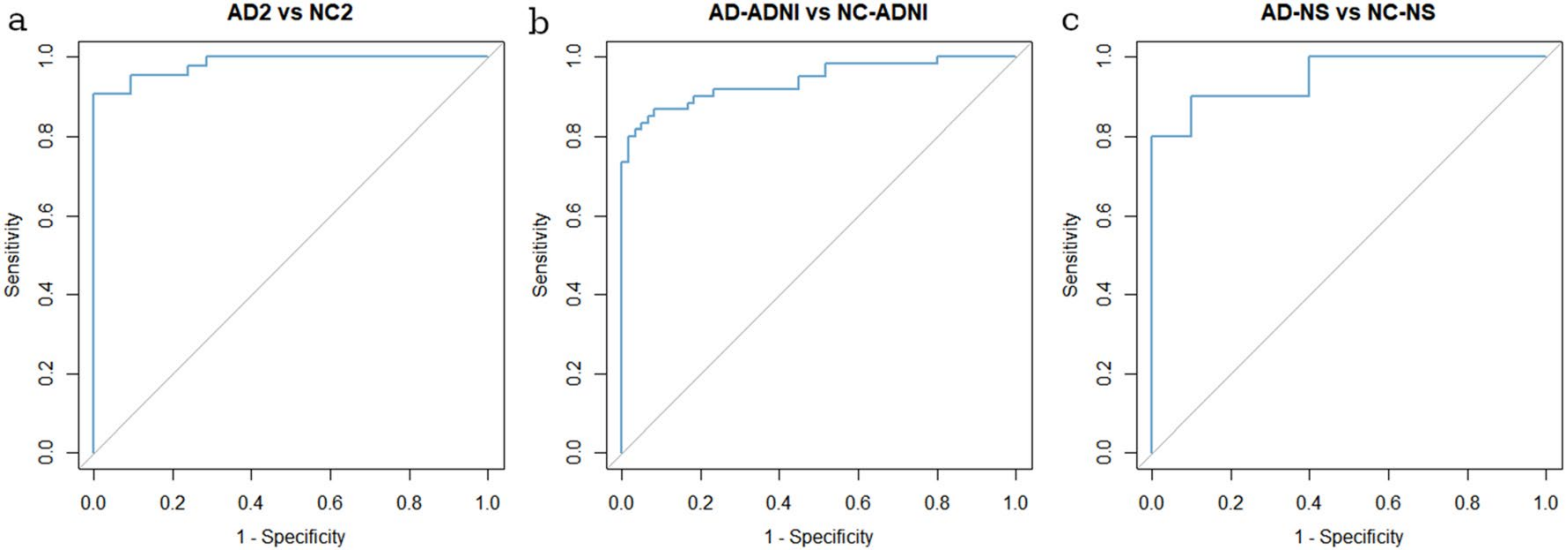
Receiver operating characteristic (ROC) curves for Alzheimer’s dementia (AD) and normal controls (NCs) on (**a**) the internal validation set, (**b**) external validation set from Alzheimer’s Disease Neuroimaging Initiative (ADNI) and (**c**) external validation set from Northshore (NS).

**Figure 5:**
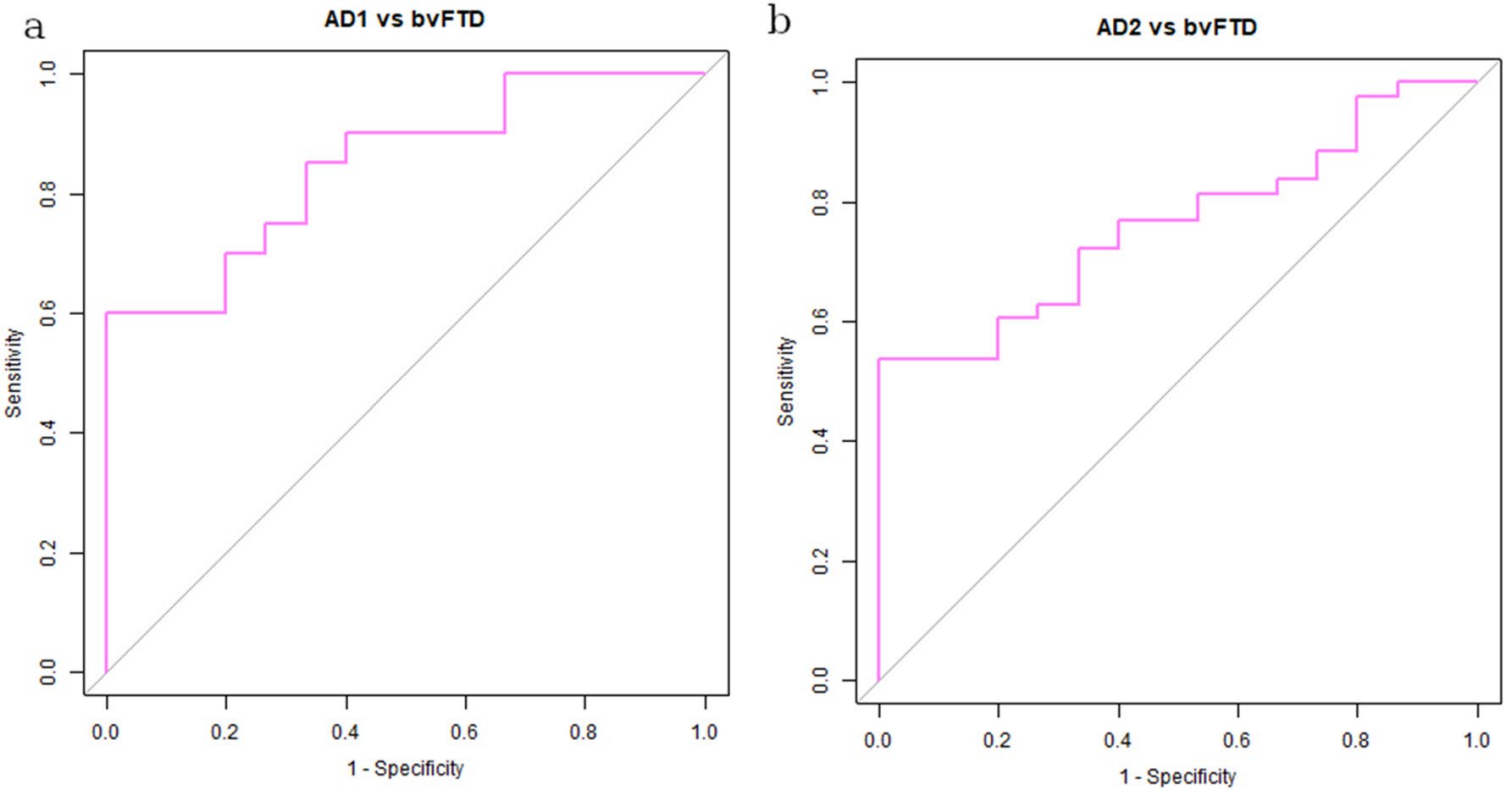
Receiver operating characteristic (ROC) curves for Alzheimer’s dementia (AD) and behavioral variant frontotemporal dementia (bvFTD) for (**a**) AD identification group (AD1) and (**b**) internal AD validation group (AD2).

There was a significant difference in ADRP expression between NC2, AD2 and FTD groups (*F*(2, 76) = 50, *p* < 0.001). Post hoc comparisons indicated that the mean subject score of AD2 group (*M* = 4.9, *SD* = 2.1) was significantly higher compared to NC2 group (*M* =  −0.1, *SD* = 1.5), *p* < 0.001, also after adjustment for age difference (*F*(1, 61) = 73, *p* < 0.001). ADRP accurately differentiated between AD2 and NC2 (AUC = 0.98, specificity = 100%, sensitivity = 91%), Fig. 4. Mean ADRP expression was significantly higher in AD2 in comparison to bvFTD group (*M* = 3.1, *SD* = 1.4), *p* = 0.004. ADRP accurately differentiated between AD1 and bvFTD (AUC = 0.85, specificity = 100%, sensitivity = 60%) and between AD2 and bvFTD (AUC = 0.76, specificity = 100%, sensitivity = 53%), Fig. 5. The post hoc comparisons also revealed significantly higher ADRP expression in bvFTD than NC2 (*p* < 0.001), Fig. 2. There was no significant difference in ADRP expression between AD1 and AD2 groups (*t*(61) = 1.69, *p* = 0.10) nor between NC1 and NC2 groups (*t*(39) = 0.28, *p* = 0.82).

**Figure 6:**
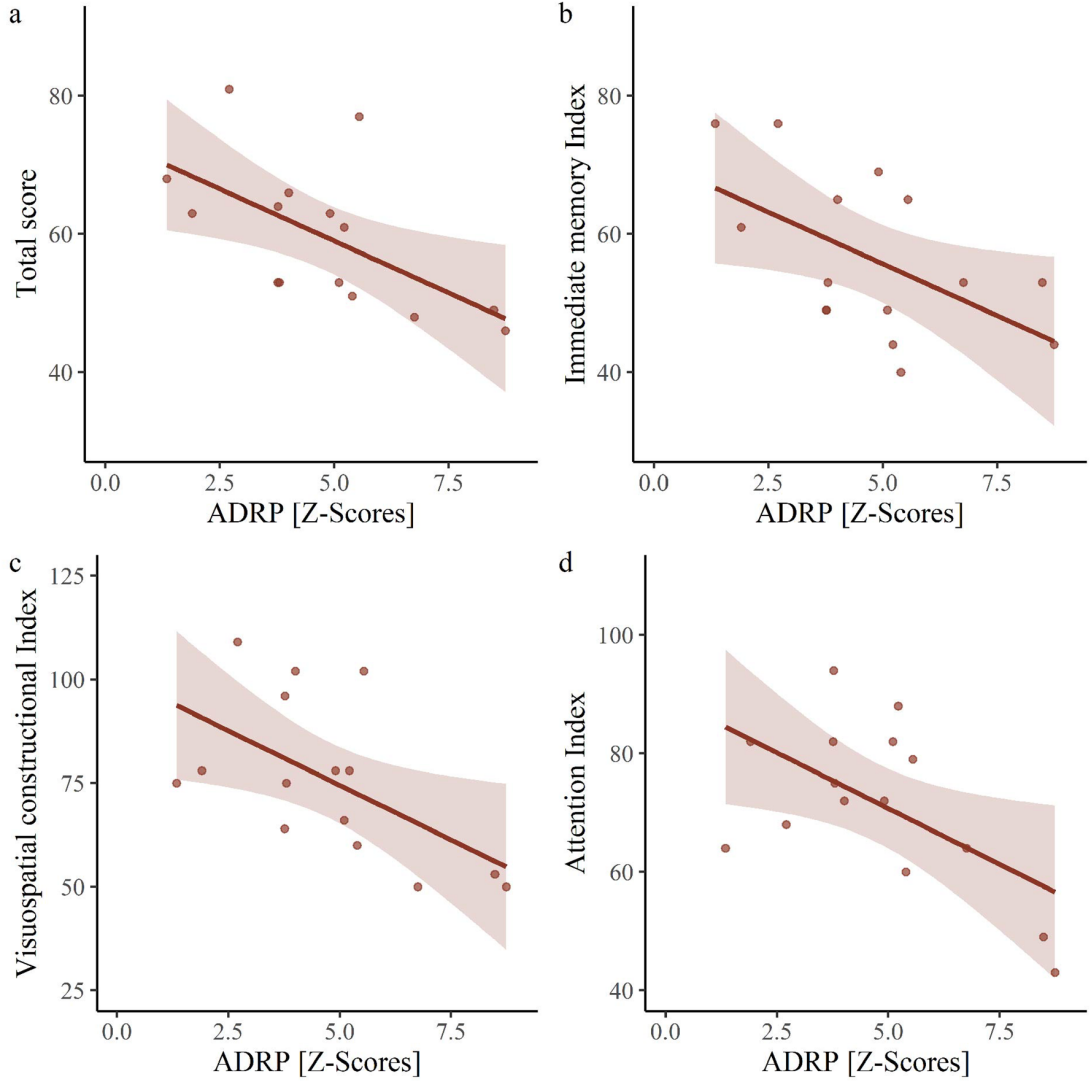
Correlations between Alzheimer’s disease-related pattern expression and neuropsychological tests in internal validation Alzheimer’s dementia cohort. Each dot represents an individual patient’s data and the lines and shaded areas correspond to the fit of a linear regression with 95% confidence intervals. (**a**) ADRP expression correlated with RBANS Total score (r =  −0.60), (**b**) Immediate Memory Index (r =  −0.55), (**c**) Visuospatial Constructional Index (r =  − 0.58) and (**d**) Attention Index (r =  −0.57). *ADRP* Alzheimer’s disease-related pattern, *RBANS* Repeatable Battery for the Assessment of Neuropsychological Status.

ADRP subject scores moderately inversely correlated with MMSE in AD2 group (*r*(37) =  −0.58, *p* < 0.001), Fig. 3a, but not with disease duration (*r*(32) =  −0.28, *p* = 0.11). ADRP expression inversely correlated also with RBANS total score (*r*(13) =  −0.60, *p* = 0.017), immediate memory Index score (*r*(13) =  −0.55, *p* = 0.034), visuospatial constructional Index score (*r*(13) =  −0.58, *p* = 0.025) and attention Index score (*r*(13) =  −0.57, *p* = 0.027), Fig. 6. ADRP subject scores did not correlate with language Index score (*r*(13) =  −0.44, *p* = 0.105) or delayed memory Index score (*r*(13) =  −0.299, *p* = 0.278).

The mean pattern expression was significantly higher in both AD1 (*M* = 5.9, *SD* = 2.5) and AD2 (*M* = 4.9, *SD* = 2.1) compared to MCI Alz group (*M* = 1.9, *SD* = 1.8), *t*(45) = 6.4, *p* < 0.001 and *t*(68) = 6.0, *p* < 0.001, respectively. ADRP expression differed significantly between NC2, MCI Alz and MCI nonAlz (*F*(2, 60) = 9.5, *p* < 0.001). It was significantly higher in MCI Alz (*M* = 1.9, *SD* = 1.8) compared to NC2 (*M* =  −0.1, *SD* = 1.5), *p* < 0.001 and also in comparison to MCI nonAlz (*M* = 0.6, *SD* = 1.6), *p* = 0.032. ADRP expression did not differ between MCI nonAlz and NC2 groups, *p* = 0.46, Fig. S1. However, we observed a significant difference in age between NC2 (*M* = 62.6, *SD* = 6.6), MCI nonAlz (*M* = 67.8, *SD* = 5.9) and MCI Alz (*M* = 73.7, *SD* = 6.1) groups (*F*(2, 60) = 19, *p* < 0.001) and after adjustment for age difference ADRP expression was no longer significantly different between the three groups (*F*(2, 59) = 1.9, *p* = 0.16), for additional information please see Supplementary material.

### External ADRP validation

We validated ADRP on two additional cohorts of AD patients and NC, i.e. ADNI and NS. ADRP expression was significantly higher in AD-ADNI (*M* = 3.8, *SD* = 1.5) than in NC-ADNI (*M* = 1.1, *SD* = 0.9) group, *t*(118) = 11.95, *p* < 0.001, Fig. 2 and it accurately differentiated between them (AUC = 0.95, specificity = 97%, sensitivity = 83%), Fig. 4. ADRP expression did not correlate with MMSE in AD-ADNI group (*r*(58) =  −0.14, *p* = 0.28), Fig. 3a, but did so with MoCA score (*r*(29) =  −0.60, *p* < 0.001), Fig. 3b. ADRP expression did not correlate with disease duration in AD-ADNI group (*r*(58) = 0.13, *p* = 0.32). ADRP expression was also significantly higher in AD-NS (*M* = 4.8, *SD* = 2.7) compared to NC-NS (*M* = 1.4, *SD* = 0.6) group, *t*(18) = 3.87, *p* = 0.001, Fig. 2 and it accurately differentiated between AD-NS and NC-NS (AUC = 0.95, specificity = 90%, sensitivity = 80%), Fig. 4. ADRP expression strongly inversely correlated with MMSE in AD-NS group (*r*(8) =  −0.85, *p* = 0.002), Fig. 3a, but not with disease duration (*r*(6) =  −0.36, *p* = 0.39).

## Discussion

In this study we newly identified an Alzheimer’s disease-related pattern—ADRP, a metabolic biomarker of AD. The ADRP is characterized by relatively reduced metabolic activity in temporoparietal cortices, posterior cingulate, thalami and precuneus which co-varied with relatively increased metabolic activity in the cerebellum. Cortical regions, associated with ADRP, have been shown previously to be involved in AD pathology. Although our understanding of the synergy between amyloid, tau and neurodegeneration remains incomplete^39^ we know that amyloid pathology begins in neocortical regions, i.e. temporal and parietal cortices and precuneus which are also part of ADRP, and later spreads to cingulate cortex and subcortical regions^40^. In numerous regions with amyloid deposits, reduction in brain metabolism has been observed^41^. Tau pathology on the other hand starts in transentorhinal cortex and affects other cortical areas only in later stages^42^. Its close correlation with hypometabolic brain changes is well known^43^. Imaging studies using either metabolic connectivity approaches or resting-state functional MRI have identified changes in similar cortical regions as comprised in ADRP^44,45^. While amyloid and tau depositions are seen in the cerebellum only in later stages^40,46^, increased metabolic activity in cerebellum, has been observed before in AD patients^12,14,16^. Cerebellum has extensive anatomical connections with the neocortex^47^, therefore its compensatory mechanism in the context of underlying pathology was proposed^48^.

ADRPs have been identified before^12,14,15^, but never in biomarker confirmed AD patients. Using a biologically heterogeneous group may have caused a lower accuracy of this biomarker in out of sample validations, AUC = 0.85–0.90^15,16^ and is indeed lower than out of sample accuracy of AUC = 0.95 achieved in our study. The topography of newly identified ADRP does significantly, but moderately, correlate (*r* = 0.51*, p* < 0.0001) with previously identified ADRP^12^, which may be caused by lack of biomarker confirmed diagnoses of AD patients in previous study, but also other factors such as different scanners or the usage of different reconstruction algorithms may have had an effect on pattern topography^49^.

We validated ADRP in two ways; statistically (i.e. bootstrapping and with threefold cross validation) and by analyzing three additional independent AD patients’ datasets. The pattern was stable on bootstrapping and we observed high correlations between PC1 of the three patterns. Further, we have shown that ADRP expression is significantly higher in AD patients compared to NC in various independent cohorts, which differentiates AD from NC with high specificity (90–100%) and sensitivity (80–91%). We paid attention to the possible effect of age on ADRP expression. Although AD patients were older than NC in the internal validation dataset, ADRP expression stayed significantly higher in AD patients after adjustment for age. Furthermore, ADRP expression did not correlate with age in any of the AD groups (*data not shown*). In addition, while the majority of our data were normally distributed, few variables in several groups (e.g., ADRP expression in the AD2 group) turned out to be non-normally distributed (*p* > 0.05, Shapiro–Wilk tests). Nonetheless, further analyses with equivalent non-parametric tests confirmed the significant results and statistical inferences of the parametric tests reported in our study.

An important measure of pattern’s clinical relevance is its correlation with subjects’ cognitive disability. ADRP expression scores correlated well with cognitive impairment in all AD groups. We observed moderate to high correlations of ADRP expression with MMSE in the identification and validation AD datasets, but not in AD cohort from ADNI database in which ADRP expression correlated with MoCA score. We believe that this may be a consequence of rather mild dementia in ADNI cohort with an average MMSE score of 23.5 (± 2). MoCA scores had a bigger range as this test is more sensitive to subtle cognitive changes^50^. Previous studies on ADRP also reported negative correlations between MMSE and ADRP expression^12,14^.

We observed a non-significant correlation of ADRP with disease duration in all AD groups. We believe that lack of correlation between ADRP expression and disease duration, which one would anticipate to be present, may be due to the insidious disease onset and difficulty of patients and caregivers in defining disease onset. Furthermore, ADRP is a biomarker of neurodegeneration, which starts when patients are still asymptomatic^41^. Previous studies did not report on this correlation. Furthermore, we did not observe any correlation between disease duration and measurements of global cognition (*r* values between −0.1 and 0.37, all *p* > 0.37).

A subset of our patients underwent a thorough neuropsychological evaluation. In these patients ADRP expression correlated significantly with indices of several cognitive domains. Negative correlations were observed with immediate memory, visuospatial constructional and attention indices which is in line with previous studies^12,14^. Both Mattis et al.^12^ and Teune et al.^14^ observed moderate correlations between ADRP expression and worse performance on tests of memory. We observed a negative correlation with immediate memory index, but not with delayed memory index. Absent correlation between ADRP expression and delayed memory index can be caused by the floor effect, since the majority of our patients scored below first percentile. ADRP expression did however correlate with tests of delayed memory in ADNI cohort where no floor effect was observed (*r*(30) =  −0.54, *p* = 0.001). Teune et al.^14^ also observed negative correlations with tests of attention, which is in line with our findings. They observed numerically similar correlations with test of visuospatial construction (*r* =  −0.55) to our study (*r* =  −0.58), although, in contrast to our finding, it did not reach statistical significance, which might be due to their smaller sample size (*n* = 11 vs. *n* = 15). While Mattis et al.^12^ observed a negative correlation with tests of executive function and Teune et al.^14^ observed non-significant correlations, the RBANS test battery, used in our study, does not contain executive function test and this correlation could not be tested in internal validation cohort. However, a significant, moderate correlation between higher ADRP expression and worse performance on Trailmaking test B was seen in ADNI cohort (*r*(34) = 0.51, *p* = 0.001). Both aforementioned studies observed significant correlations between ADRP expression and tests of language, which was not significant in our sample. This may be either due to our sample size (*n* = 15) or a variable cognitive presentation observed in AD patients, particularly in language domain^51^. Further studies, focusing on neuropsychological correlations, could offer additional insight into clinical correlations of ADRP.

Our results suggest that ADRP can be useful in differentiating between dementia syndromes. The pattern expression differentiated AD from bvFTD with high specificity, but limited sensitivity. Expression of ADRP has been previously studied in other neurodegenerative dementias. It was found that in comparison to NC, ADRP expression is higher in patients with dementia with Lewy bodies, Parkinson’s disease dementia and bvFTD^16^. Similarly, we found a higher-than-normal expression of ADRP in a cohort of bvFTD patients, which may be due to overlapping areas of neurodegeneration in these two diseases. FTD-related pattern is characterized by hypometabolic regions that are also a part of ADRP (i.e. inferior frontal, superior temporal and thalamus)^52^.

To check the performance of ADRP in earliest stages of Alzheimer’s related cognitive impairment we analyzed two groups of MCI patients: one due to Alzheimer’s disease and one due to other causes. The expression of ADRP was significantly elevated in the first compared to the latter. This trend (*p* = 0.05) remains after adjustment for age difference (see Supplementary Material for additional information).

Limitations of our study are mostly related to its retrospective design, which enabled us to analyze a large number of scans. The time difference between lumbar puncture and 2-[^18^F]FDG PET imaging varied between patients. We analyzed only patients with a maximum of 4 years (*M* = 2 months) time difference, based on previous reports on longitudinal stability of Alz CSF biomarkers at 4 years from the baseline^53,54^. Patients from different cohorts/centers were assessed by similar but not same protocols, therefore some studied subgroups are small. Furthermore, using data obtained on different scanners at different institutions could introduce unwanted data variability, which, if anything, would reduce the discriminative power of ADRP. The PET images have not been corrected for partial volume effect (PVE), which if done properly might improve ADRP performance by mitigating the regional atrophic effects. However, such analysis was beyond the scope of the current paper and the effect of PVE correction remains to be determined in future research.

## Conclusions

In our study we identified ADRP in a cohort of biomarker defined AD patients which was not done before. The precise, possibly pathologically confirmed diagnosis of the identification cohort is of utmost importance particularly when deriving a biomarker. We confirmed in this study that ADRP is a robust metabolic biomarker of AD which closely correlates with patients’ cognitive impairment. It could serve as a supportive diagnostic tool to clinicians and as a measure of specific AD-related neurodegeneration for research purposes. Its greater utility may be achieved in conjunction with specific metabolic biomarkers of other neurodegenerative dementias and by the application of novel analytical tools.

## Supplementary Information

Supplementary Information.

## Data Availability

The datasets generated during and analyzed during the current study are available from the corresponding author on reasonable request and signing a data-sharing agreement. The dataset used from ADNI repository is available at: https://adni.loni.usc.edu.
