# Supplementary material for: Identification and validation of Alzheimer’s disease-related metabolic brain pattern in biomarker confirmed Alzheimer’s dementia patients
Source: Sci Rep. 2022 Jul 11;12:11752. doi: 10.1038/s41598-022-15667-9 (PMC9273623; doi:10.1038/s41598-022-15667-9)
Supplement: Supplementary file 1 — Supplementary Information. [file 41598_2022_15667_MOESM1_ESM.docx]

# **Supplementary information**

# **for “Identification and validation of Alzheimer’s disease-related metabolic brain pattern in biomarker confirmed Alzheimer’s dementia patients”**

Matej Perovnik^1,2,*^, Petra Tomše^3^, Jan Jamšek^3^, Andreja Emeršič^4^, Chris Tang^5^, David Eidelberg^5^, the Alzheimer’s Disease Neuroimaging Initiative^6^,Maja Trošt^1,2,3^

*^1^Department of Neurology, University Medical Center Ljubljana, Zaloška cesta 2, 1000 Ljubljana, Slovenia; ^2^Faculty of Medicine, University of Ljubljana, Vrazov trg 2, 1000 Ljubljana, Slovenia; ^3^Department of Nuclear Medicine, University Medical Center Ljubljana, Zaloška cesta 2, 1000 Ljubljana, Slovenia; ^4^Laboratory for CSF diagnostics, Department of Neurology, University Medical Center Ljubljana, Zaloska cesta 2, 1000 Ljubljana, Slovenia; ^5^Center for Neurosciences, The Feinstein Institutes for Medical Research, 350 Community Drive, Manhasset, New York 11030, USA.*

***Corresponding author:** Matej Perovnik, Department of Neurology, University Medical Center Ljubljana, Zaloška cesta 2, 1000 Ljubljana, Slovenia. Email: [matej.perovnik@kclj.si](mailto:matej.perovnik@kclj.si).

1. **Supplementary Results**
   1. 3-fold cross-validation of ADRP

For the 3-fold cross-validation procedure the data from AD2 group was randomly split in two groups (*n* = 20 and *n* = 23) and SSM/PCA procedure was repeated to obtain ADRP_2 and ADRP_3. Data from NC2 was used in SSM/PCA procedure as a control group. We observed a strong correlation between ADRP_1 (characterized by the PC1 (28.7% VAF) and identified using AD1 and NC1 data), ADRP_2 (characterized as the PC1 (28.6% VAF)) and ADRP_3 (characterized as the PC1 (24.8% VAF), all *p* < 0.001, Supplementary Table S1.

**Supplementary Table S1. Correlation coefficients between patterns from cross-validation procedure.**

|  | **ADRP_1** | **ADRP_2** | **ADRP_3** |
| --- | --- | --- | --- |
| **ADRP_1** | 1.000 | 0.928 | 0.891 |
| **ADRP_2** | 0.928 | 1.000 | 0.951 |
| **ADRP_3** | 0.891 | 0.951 | 1.000 |

- 1. ADRP validation in MCI patients

Subjects’ demographic and clinical data, as well as the results of visual assessment of structural imaging and results of cerebrospinal fluid (CSF) analysis for the two mild cognitive impairment groups (MCI) are presented in Supplementary Table 1. Mean time duration between 2-[^18^F]FDG PET and lumbar puncture was 9 ± 10 months and between 2-[^18^F]FDG PET and structural imaging 13 ± 12 months in MCI groups.

There was a significant difference in age between NC2, MCI nonAlz and MCI Alz groups (*F*(2, 60) = 19, *p* < 0.001). Post hoc comparisons indicated that the MCI Alz patients were older (*M* = 73.7, *SD* = 6.1) from NC2 (*M* = 62.6, *SD* = 6.6), *p* < 0.001, and also from patients with MCI nonAlz (*M* = 67.8, *SD* = 5.9), *p* = 0.01. MCI nonAlz group was also significantly older than NC2 group, *p* = 0.04. The groups did not differ significantly in sex distribution, *p* = 0.36, but differed in MMSE (*F*(2, 44) = 6.8, *p* = 0.003) and MoCA (*F*(2,34) = 4.5, *p* = 0.02) scores. MCI Alz group had significantly lower MMSE (*p* = 0.002) and MoCA (*p* = 0.016) scores compared to NC2 group. MCI Alz group had longer disease duration (*M* = 2.8, *SD* = 1.7) than MCI nonAlz group (*M* = 1.4, *SD* = 0.9), but this difference was not statistically significant (*t*(28) = 2.06, *p* = 0.05). The two groups did not differ in MMSE (*p* = 0.16) or MoCA (*p* = 0.77) scores.

ADRP expression differed significantly between the three groups (*F*(2, 60) = 9.5, *p* < 0.001). It was significantly higher in MCI Alz (*M* = 1.9, *SD* = 1.8) compared to NC2 (*M* = −0.1, *SD* = 1.5), *p* < 0.001 and also in comparison to MCI nonAlz (*M* = 0.6, *SD* = 1.6), *p* = 0.032. ADRP expression did not differ between MCI nonAlz and NC2 groups, *p* = 0.46, Figure S1.

Due to the observed age difference between NC2, MCI nonAlz and MCI Alz groups, we have repeated analyses and included age as a covariate. Age was significantly related to the participant’s ADRP expression (*F*(1, 59) = 7.5, *p* = 0.008), but the effect of groups on ADRP expression was no longer significant after controlling for age (*F*(2, 59) = 1.9, *p* = 0.16). However, post hoc analysis of comparison of estimated marginal means based on 1000 age-stratified bootstrap samples showed that ADRP expression was significantly higher in MCI Alz group compared to NC2 group, *p* = 0.02, but not compared to MCI nonAlz group, *p* = 0.054. Estimated marginal means of ADRP expression did not differ between MCI nonAlz and NC2 groups, *p* = 0.61.

ADRP expression did not correlate significantly with MoCA scores (*r*(12) = −0.43, *p* = 0.13), nor with disease duration (*r*(21) = −0.16, *p* = 0.46) in MCI Alz group.

**Supplementary Table S2. Demographic and clinical data for MCI participants.**

|  | **NC2** | **MCI nonAlz** | **MCI Alz** | ***p* value** |
| --- | --- | --- | --- | --- |
| **N** | 21 | 15 | 27 |  |
| **Age (y)** | 62.6 (6.6) | 67.8 (5.9) | 73.7 (6.1) | < 0.001 |
| **Sex (m/f)** | 5/16 | 7/8 | 11/16 | 0.358 |
| **Disease duration (y)** | / | 1.4 (0.9) | 2.8 (1.7) | 0.050 |
| **MMSE** | 29.4 (0.8)  (*n* = 12) | 28.2 (1.2)  (*n* = 14) | 27 (2.4)  (*n* = 21) | 0.003 |
| **MoCA** | 27.6 (1.7)  (*n* = 13) | 25.5 (2.5)  (*n* = 10) | 24.8 (3.1)  (*n* = 14) | 0.018 |
| **MTA score (left+right)** | / | 2 (1.3)  (*n* = 3) | 2.8 (1.4)  (*n* = 13) | 0.391 |
| **Fazekas** | / | 1.3 (1.5)  (*n* = 3) | 1.0 (1.0)  (*n* = 13) | 0.677 |
| **Alz CSF (positive/total)** | 0/3 | 0/15 | 27/27 |  |

^Data is presented as mean (SD). NC2 – normal controls validation group. MCI nonAlz – mild cognitive impairment due to other causes. MCI Alz – mild cognitive impairment due to Alzheimer’s disease. MMSE – Mini Mental State Examination. MoCA – Montreal Cognitive Assessment. MTA – medial temporal lobe atrophy. CSF – cerebrospinal fluid. Positive Alzheimer (Alz) CSF was defined as A+/T+/N+ or A+/T+/N− (cut-offs: Aβ42 < 650 pg/ml or Aβ42/Aβ40 < 0.077, p-tau > 60 pg/ml, t-tau >400 pg/ml).^

**
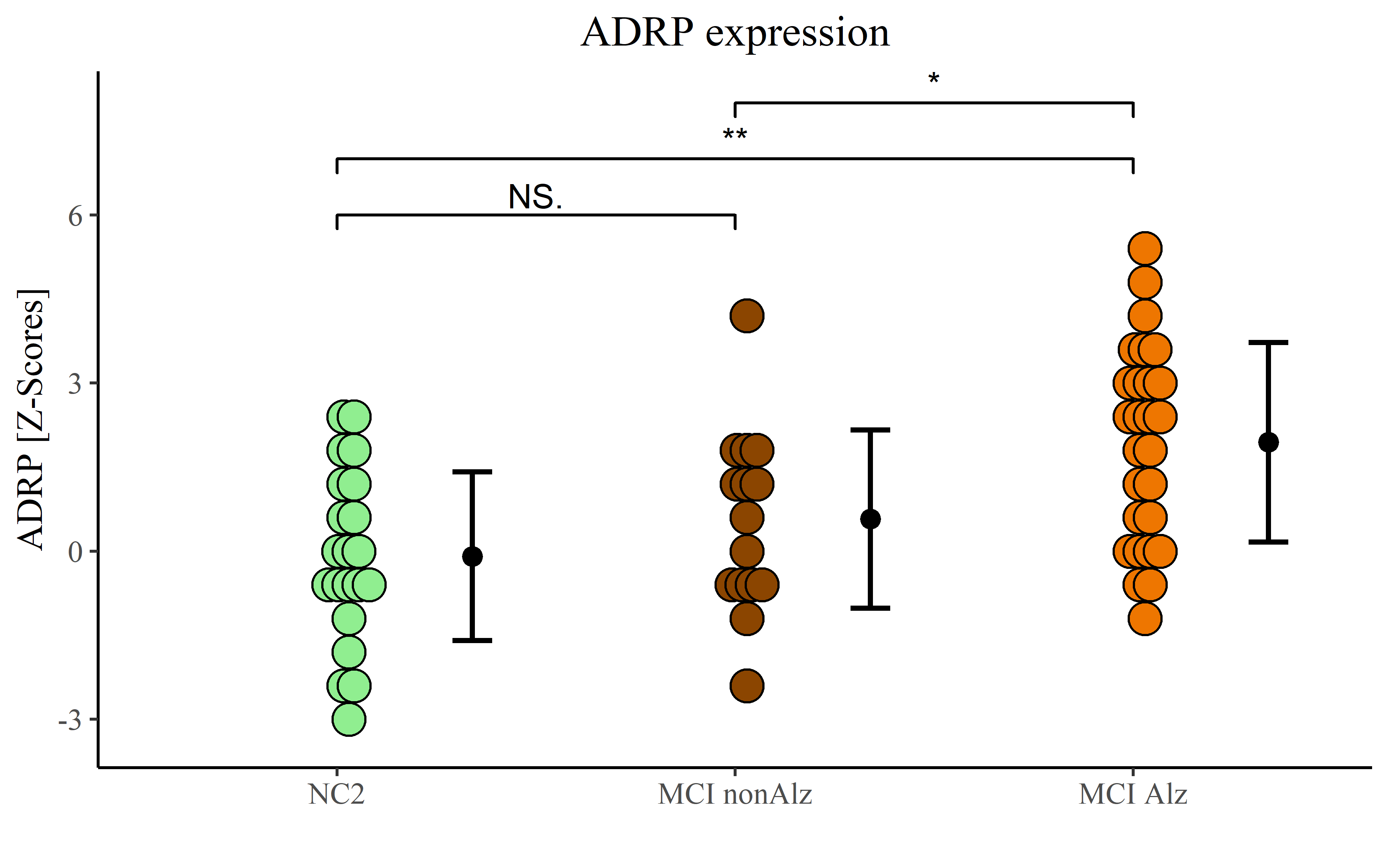
**

**Figure S1** Alzheimer’s disease-related pattern expression in MCI groups. Data are Z-scored based on the pattern expression in NC identification group. Means (SD) are presented to the right of individual data. ADRP – Alzheimer’s disease-related pattern. NC2 – normal controls validation group. MCI nonAlz – MCI due to other causes. MCI Alz – mild cognitive impairment due to Alzheimer’s disease. **p < 0.001, *p < 0.05, NS – non-significant

1. **Supplementary Discussion**

Making the correct diagnosis in the early stages of neurodegenerative syndromes may be challenging for clinicians. Correct diagnosis and the evaluation of the disease stage is of great importance for research. Therefore, we tested the performance of ADRP in differentiating two groups of MCI patients, one due to Alzheimer’s disease and the other due to other causes. Expression of ADRP was significantly elevated in patients with MCI due Alzheimer’s disease, but not in patients with MCI due to other causes, even though the two groups did not differ in their MMSE or MoCA scores. However, after the adjustment for age difference this result was no longer significant. We believe though that the difference in ADRP expression between MCI Alz and MCI nonAlz group is not driven only by the age difference, but that the loss of significance is caused by relatively small number of subjects (*n* = 15) in MCI nonAlz group. Previous studies have shown some, but not consistent, correlation between ADRP and age in various cohorts ^1–6^. Normal ageing affects brain areas that are part of the ADRP topography ^7^ therefore the relationship between ageing and ADRP need to be specifically addressed in future research.

# **References**

1. Scarmeas, N. *et al.* Covariance PET patterns in early Alzheimer’s disease and subjects with cognitive impairment but no dementia: utility in group discrimination and correlations with functional performance. *Neuroimage* **23**, 35–45 (2004).

2. Habeck, C. *et al.* Multivariate and univariate neuroimaging biomarkers of Alzheimer’s disease. *Neuroimage* **40**, 1503–1515 (2008).

3. Teune, L. K. *et al.* The Alzheimer’s disease-related glucose metabolic brain pattern. *Curr Alzheimer Res* **11**, 725–32 (2014).

4. Mattis, P. J. *et al.* Distinct brain networks underlie cognitive dysfunction in Parkinson and Alzheimer diseases. *Neurology* **87**, 1925–1933 (2016).

5. Meles, S. K. *et al.* The Alzheimer’s disease metabolic brain pattern in mild cognitive impairment. *J. Cereb. Blood Flow Metab.* **37**, 3643–3648 (2017).

6. Katako, A. *et al.* Machine learning identified an Alzheimer’s disease-related FDG-PET pattern which is also expressed in Lewy body dementia and Parkinson’s disease dementia. *Sci. Rep.* **8**, 1–13 (2018).

7. Ishibashi, K. *et al.* Longitudinal effects of aging on 18F-FDG distribution in cognitively normal elderly individuals. *Sci. Rep.* **8**, 11557 (2018).
